# Tadpole system as new lumbar spinal instrumentation

**DOI:** 10.1186/1749-799X-3-41

**Published:** 2008-09-12

**Authors:** Yuichi Kasai, Tadashi Inaba, Koji Akeda, Atsumasa Uchida

**Affiliations:** 1 Department of Orthopaedic Surgery, Mie University Graduate School of Medicine, 2-174 Edobashi, Tsu city, Mie prefecture, 514-8507, Japan; 2 Department of Mechanical Engineering, Mie University, Tsu city, Mie prefecture, Japan

## Abstract

**Background:**

There have been reports of serious complications associated with pedicle screw fixation, including nerve root injuries caused by accidental screw insertion. We have developed a new system of lumbar spinal instrumentation that we call Tadpole system^®^. The purposes of this report were to show the results of a biomechanical study and the short-term outcome of a clinical study, as well as to determine the usefulness of this system.

**Methods:**

The Tadpole system^® ^lumbar spinal fusion is a hook-and-rod system according to which the spine is stabilized using 2 sets of 2 spinous processes each that are held in place by 4 hooks tandemly connected to a rod. The biomechanical study was done using 5 human lumbar cadaveric spines, and the range of motion (ROM) was examined in a non-treatment model, an injured model, a pedicle screw fixation model and a Tadpole system^® ^model. For the short-term clinical study the Tadpole system^® ^was used in 31 patients, and the factors analyzed were operation time, time required for spinal instrumentation, amount of intraoperative bleeding, postoperative improvement rate of the Japanese Orthopaedic Association (JOA) score for lumbar spinal disorders, instrumentation failure, spinous process fracture, spinal fluid leakage, nerve root injury, postoperative infection, and bone fusion 2 years after the operation.

**Results:**

The ROM in the Tadpole system^® ^model was slightly bigger than that in the pedicle screw fixation model, but smaller than that in the normal control model. These biomechanical data indicated that the Tadpole system^® ^provided fairly good stability. The mean operation time was 79 min, the mean time required for spinal instrumentation was 8 min, and the mean amount of intraoperative bleeding was 340 mL. The mean postoperative improvement rate of JOA score was 70.9 ± 24.8%. Instrumentation failure (dislocation of a hook) occurred in one patient, and none of the patients developed spinous process fracture, spinal fluid leakage, nerve root injury, or postoperative infection. Two years after the operation, bone union was confirmed in 29 of the 31 patients (93.5%).

**Conclusion:**

We conclude that this system is a useful, easy-to-use and safe spinal instrumentation technique for lumbar fusion surgery.

## Background

Lumbar spinal instrumentation has been widely used for pedicle screw fixation (PSF), with generally favorable clinical outcomes. However, there have been reports of serious complications associated with this method, including nerve root injuries caused by accidental screw insertion [1,2]. We have developed a new system of lumbar spinal instrumentation, which we call Tadpole system^® ^(Kisco DIR Co., Ltd., Osaka, Japan), that uses the spinous processes as anchors.

In the present report, we describe the Tadpole system^®^, the results of a biomechanical study and the short-term outcome of a clinical study.

### Tadpole system

#### Overview

**Figure 1:**
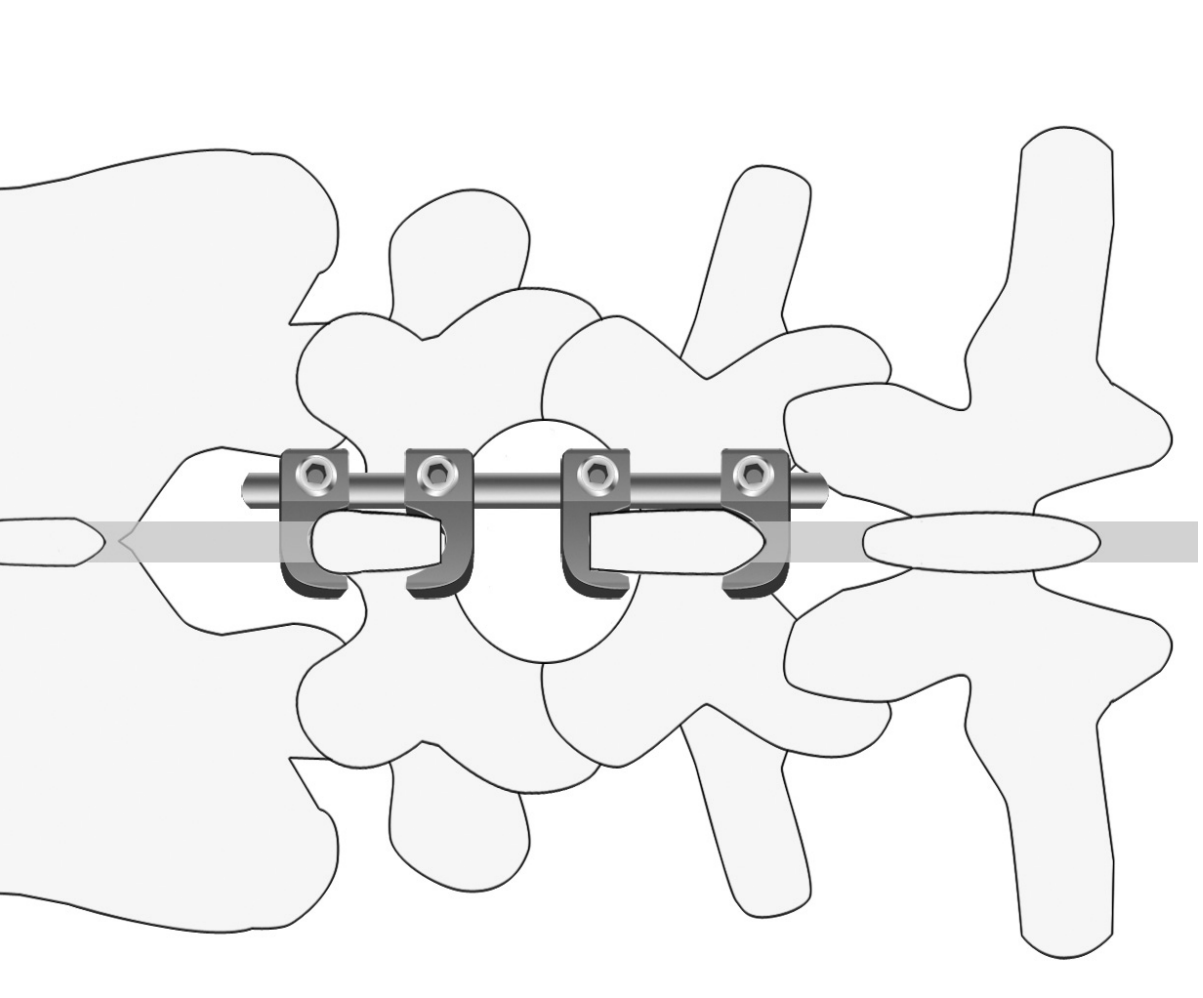
Tadpole system^®^; hook-and-rod system of spinal instrumentation.

The Tadpole system^® ^is used in spinal fusion to treat lumbar spinal canal stenosis and lumbar degenerative spondylolisthesis. It is a hook-and-rod system of spinal instrumentation in which the spine is stabilized using 2 sets of 2 spinous processes each that are held in place by 4 hooks tandemly connected to a rod (Figure 1). The hooks are 7 to 15 mm in length, and the rods are 4 to 12 cm in length. The hook is connected to the rod by tightening a nut that is attached to the hook. Because the hook resembles a tadpole, we named this system "Tadpole".

**Figure 2:**
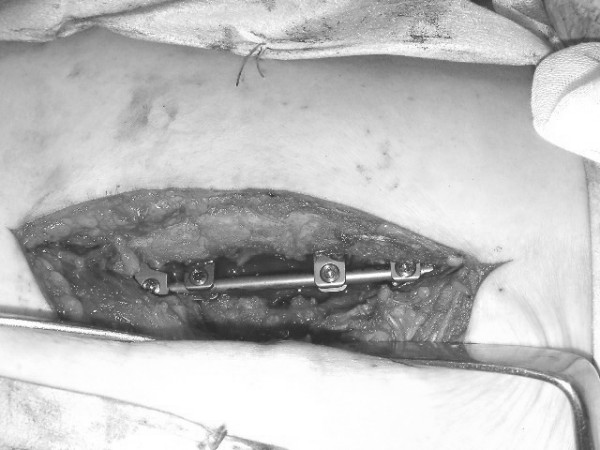
Tadpole system^® ^positioned using a unilateral approach.

**Figure 3:**
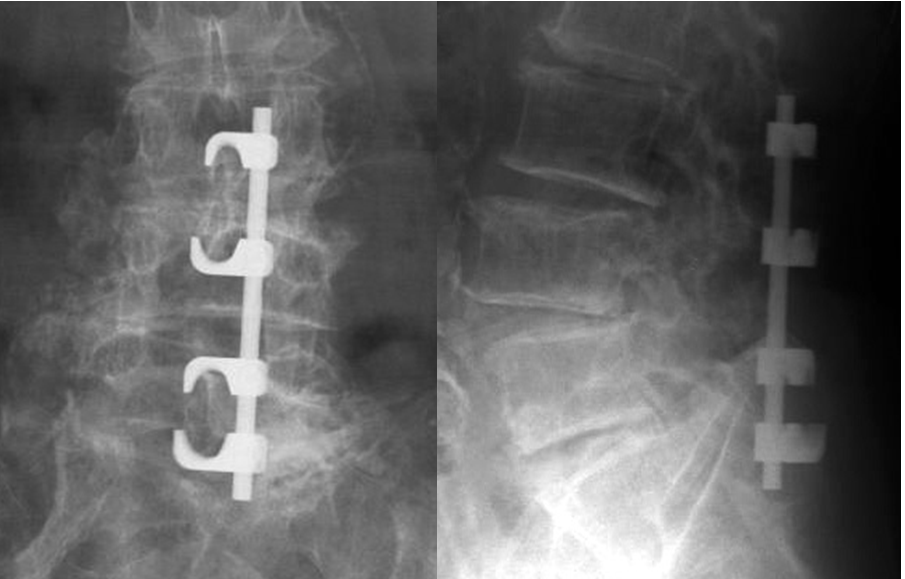
X-rays of a case of two-level fusion

**Figure 4:**
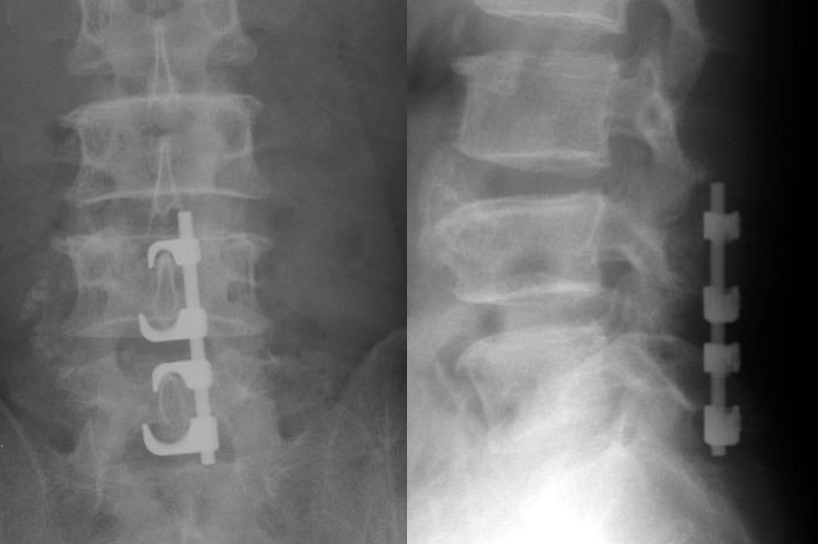
X-rays of a case of single-level fusion

With the Tadpole system^®^, implants can be positioned using a unilateral approach (Figure 2), and a sufficient base for posterolateral fusion can be retained due to its placement toward the median line. This system is commonly used for two-level fusion (Figure 3) between L3 and L5 or between L2 and L4, or for single-level fusion (Figure 4) between L3 and L4 or L4 and L5. However, this system is not applicable for fusions that include the sacral spine, because the spinous process of S1 is too small to be fixed with the hook. It is neither applicable in patients with spondylolysis or severe spinal instability, nor for correction of spinal alignment.

#### Operative techniques when using the Tadpole system^®^

Surgery is performed with the patient in the prone position and under general anesthesia. The paravertebral muscles are disclosed after a posterior median incision of the lumbar spinal region. Laminectomy or spinal decompression by fenestration is then performed. If necessary, transforaminal lumbar interbody fusion (TLIF) is performed with interbody cages before using the Tadpole system^®^. It is important to minimize resection of the spinous processes, because excessive resection may result in their fracture or increased instability of the hook. In particular, an excessive cut of the spinous process of L5 should be avoided because this is frequently smaller than that of L3 and L4.

**Figure 5:**
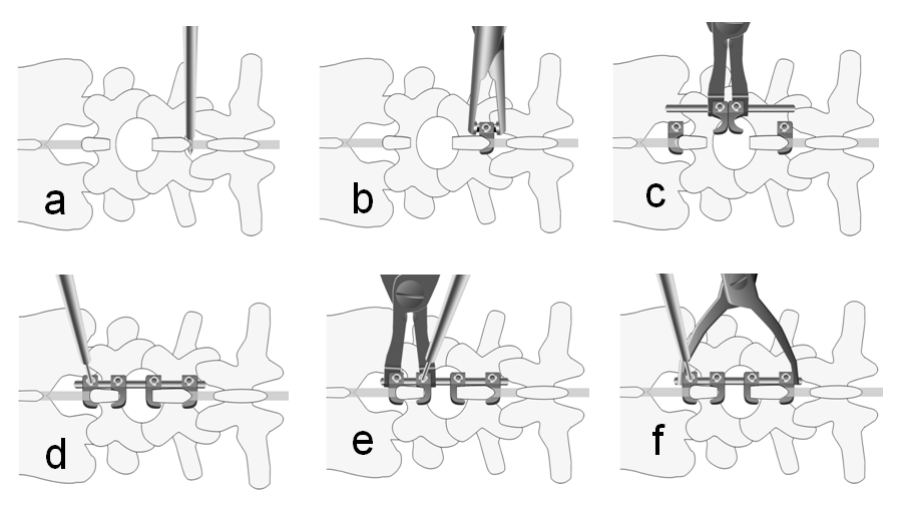
**Operative techniques for Tadpole system®**  a; Guide holes for hooks are made on the interspinous ligament using a starter awl b; The hook is held with a hook holder, and it is inserted into the interspinous ligament c; The 2 hooks are connected in the center to a rod, and the rod is connected to the cranial and caudal hooks. d; The hooks are tightened temporarily while compression is maintained between the cranial and caudal hooks. e; The set of 2 hooks for the stabilization of a spinous process is tightened temporarily, pinching each spinous process f; The screws of all hooks are tightened using a screwdriver

After spinal decompression, guide holes for hooks are made on the interspinous ligament using a starter awl (Figure 5A; in this case holes were made between L3 and L4, and between L5 and S1). The size of the hook is then determined using a frontal-view preoperative plain lumbar radiograph. The cranial and caudal hooks are held with a hook holder and inserted into the interspinous ligament (Figure 5B), and then the stability of the hooks in the interspinous ligament should be confirmed.

Next, 2 hooks are connected in the center to a rod, and the rod is connected to the previously inserted cranial and caudal hooks while holding the 2 hooks with a hook holder (Figure 5C). The hooks are tightened temporarily while compression is maintained between the cranial and caudal hooks (Figure 5D). It is important not to apply excessive compression to avoid a spinous process fracture. Thereafter, the 2 hooks for the stabilization of each spinous process are tightened temporarily, pinching each spinous process (Figure 5E). Finally, the screws of all hooks are tightened using a screwdriver (Figure 5F).

In patients who do not require TLIF treatment, the superior articular process of the portion to be fixed, the lateral portion of the intervertebral joint, and the base of the transverse process should be completely decorticated, and posterolateral fusion should be implemented using a removed piece of the ilium or a local bone.

### Biomechanical study using the Tadpole system^®^

#### Method

The specimens used were 5 human lumbar cadaveric spines consisting of L2 to L5 removed from donor bodies, which were autopsied in our university hospital; the specimens were used with the consent of the donors' family members. Both ends of each lumbar spine were mounted with dental resin and set on a moment-loading instrument. Four experimental models were made step by step from each lumbar spine. The experimental models included the non-treatment model (normal control model), an injured model (with resection of the lateral facet joint between L3 and L4), a PSF model generated from the injured model with pedicle screw fixation (Absorbing Shock Device^®^, KiscoMedica, Saint Priest, France), and a Tadpole system^® ^(TS) model created from the injured model. A marker was placed on the vertebral bodies of L3 and L4, and a moment load of 5 N.m was applied to each experimental model for forward flexion, backward flexion, left side bending, right side bending, left rotation, and right rotation. The movement of the markers on the vertebral bodies of L3 and L4 after application of a moment load and with no load was photographed with a digital camera. The ROM for backward and forward flexion, right and left side bending, and right and left rotation between L3 and L4 was determined using a Microsoft Office Visio^® ^image processing software.

#### Results

The mean ROM of the five cadaveric spines is shown in Table 1. The ROM for all backward and forward flexions and right and left side bending was smaller in the TS model and PSF model than in the injured model. The ROM in the TS model was slightly bigger than that in the PSF model, but smaller than that in the normal control model. These data indicated that the Tadpole system^® ^provided fairly good stability.

**Table 1:** Mean range of motion of each model on the biomechanical study

			(mean± SD)
	ROM of forward – backward flextion	ROM of right – left side bending	ROM of right – left side rotaton
Normal control model	7.2° ± 4.8°	7.8° ± 3.8°	13.8° ± 7.1°
Injured model	18.6° ± 6.2°	22.1° ± 8.8°	24.8° ± 10.4°
PSF model	4.3° ± 2.9°	2.5° ± 1.3°	3.4° ± 2.6°
TS model	3.4° ± 2.2°	5.1° ± 2.5°	7.9° ± 5.2°

Normal control model; non-treatment model

Injured model; model resected the facet joint between L3 and L4

PSF model; model generated from the injured model with pedicle screw fixation

TS model; model created from the injured model with the Tadpole system^®^

### Short-term clinical study using the Tadpole system^®^

#### Subjects and methods

The subjects were 31 patients (19 men and 12 women) who underwent spinal fusion using the Tadpole system^® ^and were followed up ≥ 2 years in our clinic. Twenty-seven patients had lumbar spinal canal stenosis and 4 had spondylolisthesis. Intervertebral fusion of L2–L4, L3–L5, L3–L4 and L4–L5 was performed in 4, 22, 1 and 4 patients, respectively; 8 of the 31 patients underwent surgery via a unilateral approach, and all patients underwent posterolateral fusion. The mean age at the time of the operation was 73.3 years (range: 59 to 84 years). The mean follow-up period was 2 years 3 months (range: 2 years to 2 years 9 months).

The factors analyzed in this study were operation time, time required for spinal instrumentation, amount of intraoperative bleeding, Japanese Orthopaedic Association (JOA) score for lumbar spinal disorders on a 29-point scale before and 2 years after the operation, postoperative improvement rate of JOA score (%, Hirabayashi method), instrumentation failure, spinous process fracture, spinal fluid leakage, nerve root injury, postoperative infection, and bone fusion 2 years after the operation. Evaluation of lumbar spinal fusion is partially subjective, therefore, the X-ray images were assessed by two independent observers and it was determined that fusion had been achieved when both observers confirmed there was no pseudoarthrosis. Pseudoarthrosis was diagnosed when any gap in the fusion mass on antero-posterior or oblique radiographs was seen, or if there were more than two degrees of motion on flexion-extension films. The rate of consistency of fusion status grading achieved by the two observers was 100%, and interjudge reliability was very good.

## Results

The mean operation time was 79 ± 41 min (± S.D.), the mean time required for spinal instrumentation was 8 ± 3 min, and the mean amount of intraoperative bleeding was 340 ± 278 mL. The mean JOA score was 14.2 ± 7.1 points before the operation, and 24.7 ± 3.8 points after the operation; the mean postoperative improvement rate was 70.9 ± 24.8%. Dislocation of a hook occurred in one patient, however, this patient showed solid spinal fusion one year after the surgery. None of the patients developed spinous process fracture, spinal fluid leakage, nerve root injury, or postoperative infection. Two years after the operation, bone union was confirmed in 29 of the 31 patients (93.5%).

## Discussion

Although clinical studies have generally shown favorable outcomes for lumbar fusion surgery using pedicle screw fixation, there have been reports of complications including spinal fluid leakage (4%), nerve injury (2%), deep infection (4–5%), and instrumentation failure (3 – 12%) [1,2]. Another disadvantage of this technique is radiation exposure of operators and patients, because X-ray images are taken during the surgery.

Among several methods of posterior spinal instrumentation, the use of the spinous processes as anchors provides less biomechanical strength than pedicle screw fixation [3], but produces no complications such as spinal fluid leakage or nerve injury, and requires no excessive excision of the paraspinal muscles [4]. In addition, the relatively low invasiveness of spinal instrumentation using the spinous processes is a key advantage, because such method is associated with shortened operation time, reduced bleeding, and reduced length of subsequent hospital stay. Thus, the Tadpole system^® ^we have developed, which is an easy-to-perform fixation technique that is less invasive than other techniques, may become widely accepted and can be expected to result in good cost-effectiveness.

Spinal instrumentation using the spinous processes as anchors has been performed for approximately 50 years [5,6]. In case studies, the Daab plate and the Wilson plate have been used, and large-scale studies have not been conducted as yet. The pull-out strength of the spinous process wiring technique developed by Drummond et al. [7] was reported to be 30% to 45%, compared with sublaminar wiring. This suggests that sublaminar wiring is preferable to spinous process wiring [3]. Coe et al. [8] reported that the fracture load of the spinous process is one-fifth to one-half of that of the vertebral arch. Because the biomechanical strength of the spinous process is not high, there is limited flexibility in spinal instrumentation using the spinous process as an anchor, but recent studies of lumbar fusion surgery using the Lumbar Alligator Spinal system^® ^[9] and CD Horizon Spire spinous process plate^® ^[4,10] have shown relatively favorable clinical outcomes. In a biomechanical study carried out by Shepherd et al. [11], holding the spinous process with a hook provided sufficient holding ability. Thus, the available evidence indicates that the Tadpole system^® ^has advantages over other systems.

The mean intervertebral fusion rate in the present study was as high as the 96% attained with lumbar fusion surgery using pedicle screw fixation [12-14]. The Lumbar Alligator Spinal system^® ^with spinal instrumentation using the spinous process as an anchor was associated with an intervertebral fusion rate of 92.7% (104 of 107 patients) [9], and the intervertebral fusion rate of our Tadpole system^® ^was 93.5%, which is quite good.

In future studies, we plan to: 1) use data from long-term follow-up clinical studies; 2) increase the number of study patients; 3) use clinical data for a unilateral approach; 4) examine postoperative changes in vertebral bodies adjacent to fixed vertebrae; and 5) attempt to establish the criteria for deciding between pedicle screw fixation, the Tadpole system^®^, and no spinal instrumentation.

## Conclusion

The clinical outcomes of the Tadpole system^® ^were generally favorable. Therefore, we conclude that this system is a useful, easy-to-use and safe spinal instrumentation technique for lumbar fusion surgery.

## Competing interests

Yuichi Kasai, the inventor of Tadpole system, receives royalities from Kisco DIR Co., Ltd. resulting from its sale.

## Authors' contributions

YK and TI have made substantial contributions to conception and design, or acquisition of data, or analysis and interpretation of data. YK and KA have been involved in drafting the manuscript or revising it critically for important intellectual content. AU has given final approval of the version to be published.
